# The causal effect and impact of reproductive factors on breast cancer using super learner and targeted maximum likelihood estimation: a case-control study in Fars Province, Iran

**DOI:** 10.1186/s12889-021-11307-5

**Published:** 2021-06-24

**Authors:** Amir Almasi-Hashiani, Saharnaz Nedjat, Reza Ghiasvand, Saeid Safiri, Maryam Nazemipour, Nasrin Mansournia, Mohammad Ali Mansournia

**Affiliations:** 1 Department of Epidemiology, School of Health, Arak University of Medical Sciences, Arak, Iran; 2 Traditional and Complementary Medicine Research Center, Arak University of Medical Sciences, Arak, Iran; 3 Department of Epidemiology and Biostatistics, Knowledge Utilization Research Center, School of Public Health, Tehran University of Medical Sciences, Tehran University of Medical Science, Tehran, Iran; 4 Department of Research, Cancer Registry of Norway, Oslo, Norway; 5 Oslo Centre for Biostatistics and Epidemiology, Oslo University Hospital, Oslo, Norway; 6 Aging Research Institute, Tabriz University of Medical Sciences, Tabriz, Iran; 7 Department of Community Medicine, Faculty of Medicine, Tabriz University of Medical Sciences, Tabriz, Iran; 8 Osteoporosis Research Center, Endocrinology and Metabolism Clinical Sciences Institute, Tehran University of Medical Sciences, Tehran, Iran; 9 Psychosocial Health Research Institute, Iran University of Medical Sciences, Tehran, Iran; 10 Department of Endocrinology, AJA University of Medical Sciences, Tehran, Iran; 11 Department of Epidemiology and Biostatistics, School of Public Health, Tehran University of Medical Sciences, P.O Box: 14155-6446, Tehran, Iran

**Keywords:** Breast neoplasms, Reproductive history, Case-control study, Population attributable fraction, Causal analysis, Double robustness, TMLE, Super learner

## Abstract

**Objectives:**

The relationship between reproductive factors and breast cancer (BC) risk has been investigated in previous studies. Considering the discrepancies in the results, the aim of this study was to estimate the causal effect of reproductive factors on BC risk in a case-control study using the double robust approach of targeted maximum likelihood estimation.

**Methods:**

This is a causal reanalysis of a case-control study done between 2005 and 2008 in Shiraz, Iran, in which 787 confirmed BC cases and 928 controls were enrolled. Targeted maximum likelihood estimation along with super Learner were used to analyze the data, and risk ratio (RR), risk difference (RD), andpopulation attributable fraction (PAF) were reported.

**Results:**

Our findings did not support parity and age at the first pregnancy as risk factors for BC. The risk of BC was higher among postmenopausal women (RR = 3.3, 95% confidence interval (CI) = (2.3, 4.6)), women with the age at first marriage ≥20 years (RR = 1.6, 95% CI = (1.3, 2.1)), and the history of oral contraceptive (OC) use (RR = 1.6, 95% CI = (1.3, 2.1)) or breastfeeding duration ≤60 months (RR = 1.8, 95% CI = (1.3, 2.5)). The PAF for menopause status, breastfeeding duration, and OC use were 40.3% (95% CI = 39.5, 40.6), 27.3% (95% CI = 23.1, 30.8) and 24.4% (95% CI = 10.5, 35.5), respectively.

**Conclusions:**

Postmenopausal women, and women with a higher age at first marriage, shorter duration of breastfeeding, and history of OC use are at the higher risk of BC.

**Supplementary Information:**

The online version contains supplementary material available at 10.1186/s12889-021-11307-5.

## Highlights

- ✓ Postmenopausal women, women with higher age at marriage, women with lower breastfeeding duration, and women with a history of OC usage are at greater risk of BC.
- ✓ The most important risk and preventive factors were menopausal status and breastfeeding history, respectively.
- ✓ Encouraging people to marry at a younger age, and increasing breastfeeding duration, as well as policies to reduce the use of hormonal contraceptives, can be effective in reducing BC cases.

## Introduction

In previous decades, most cases of cancer have occurred in more developed countries, but recently the pattern has shifted towards developing or less-developed countries; these countries account for about 82% of the world’s population, with about 57% of cancer cases and 65% of deaths from cancer [1]. According to GLOBOCAN, about 18.1 million new cases of cancer and 9.6 million deaths from cancer were reported in 2018 [2].

Breast Cancer (BC) has the highest number of incident cases [3]; it alone accounts for 25% of cancer cases and 15% of cancer deaths among women, with almost half of the new cases and 38% of deaths occurring in more developed countries [1, 4]. It has the highest annual incidence among women in 161 countries and is also the leading cause of cancer death in 98 countries [5]. With 1.68 million cases in 2016, BC was reported as the most common cancer among women, with 535,000 deaths and 15.1 million DALYs [6]. About one-third of new cases of cancer among women is BC [7]. In recent years, the incidence and deaths from BC have increased in Asian countries including Iran [1, 3, 6].

Several factors, such as smoking, being overweight, screening programs, physical inactivity, and changes in reproduction patterns associated with urbanization and economic development have contributed to the increase in the incidence of BC [1, 2, 8]. Several studies have been carried out on the role of reproductive factors and contradictory results have been reported [9–16].

The causal study of the risk factors of BC requires careful adjustment for confounders. There are two broad approaches for confounding adjustment: conventional outcome regression modeling and propensity score methods (exposure modeling) [17]. The double-robust approach combines outcome and exposure models [18]. Misspecification of regression models may cause extreme bias in treatment effect estimates. This problem has led to a growing interest in using adaptive regression techniques, such as machine learning methods in causality research [19–22]. In particular, the field of targeted learning has emerged as a paradigm for wedding machine learning and formal statistical inference [23].

There are many methods for the causal analysis of case-control data, such as inverse probability-of-treatment weighting (IPTW), parametric g-formula (model-based standardization), and targeted maximum likelihood estimation (TMLE), all of which estimate the so-called marginal (population-averaged) causal effects [24–38]. The TMLE method is a combination of the IPTW and parametric g-formula and so is double-robust: there are two possibilities for correct model specification [29].

The relationship between reproductive factors and risk of BC has been investigated in previous studies, but there were discrepancies in the reported results. Also to our best of knowledge, the causal effects of reproductive factors on BC have not been studied. Therefore, the aim of this study was to estimate the causal effect of reproductive factors on the risk of BC in a case-control study using TMLE [39] and Super Learner algorithms [39, 40] to adjust for confounders.

## Materials and methods

### Study design

In this case-control study, frequency matching was performed by age, with five-year intervals. BC cases were confirmed by histopathology and their data were collected in the Cancer Registry Center of Shiraz University of Medical Sciences. This study was designed in 2005 and data were collected between September 2005 and December 2008 in Shiraz, Iran, and the available data were re-analyzed in 2018 (as PhD dissertation of the first author) in order to achieve more valid estimates, applying the advanced causal methods. The case data were collected from the main hospitals in Shiraz, covering over 85% of the incident cases in the city. In this study, more than 93% of the subjects were interviewed within a maximum of 6 months after diagnosing BC. The control group was selected from the Faqihi Hospital (as a general hospital) in Shiraz and from women without a history of BC or diseases with common risk factors with BC (such as gynecology, neoplasm, and hormonal disorders, and those referred to the skin clinic, internal medicine, and urology). Only participants with complete information (787 cases and 928 controls) for all variables were included. The study was approved in Tehran University of Medical Sciences (Code: 9121128009) in terms of methodology also by the Ethical Committee of Shiraz University of Medical Sciences (project number 591–2). All participants provided informed consent to be included in the study. Further details on the study design have been published [12, 41]. All methods were performed in accordance with the approved protocol as well as STROBE guideline.

### Data gathering

Variables were collected through interviewing by two nurses trained in the same way so that there is no heterogeneity in the data collection. A checklist (including socioeconomic, demographic, and reproductive factors) was used to collect relevant variables for BC.

### Variables

Reproductive factors were identified as potential exposures, and anthropometric and socioeconomic factors as potential confounders. Reproductive variables, including parity (≤3, > 3), menopausal status (post-, pre-menopausal), age at first pregnancy (< 25, ≥25 years old), age at first marriage (< 20, ≥20 years old), history of breastfeeding duration (≤60, > 60 months) and history of oral contraceptive (OC) use (ever, never), were considered as exposure variables and BC as the outcome.

Causal directed acyclic graphs (DAGs) [42–45] were used to identify the minimally sufficient set of confounders for effect of each exposure on the outcome (Supplementary Figures S1, S2, S3, S4, S5 and S6). In order to simplify the DAGs without loss of the validity of the back-door criterion, we avoided to present some arrows between covariates that did not play a role in identifying confounders. The causal relationship between variables (the arrows) was determined based on our prior knowledge and review of literature. The selection of which individuals to study (sampling) was influenced by their age (matched variables) and their disease status (case and control group), shown in the diagrams with arrows. In the figures, the variable S indicates selection of people from the hypothetical cohort into this case-control study (1:selected, 0: not selected). The arrows from BC and age to S reflect the age-frequency-matched case-control selection, and rectangle surrounding S = 1 indicates analysis is conditional on the selected individuals [46–48].

### Statistical analysis

We used TMLE method to estimate the causal effect of reproductive factors on BC. We estimated marginal risk difference (RD) and risk ratio (RR) as well as population attributable fraction (PAF) for the BC risk factors. We used a modification of TMLE appropriate for analyzing of case-control data, case-control weighted targeted maximum likelihood estimation (CCW-TMLE) [29, 30]. Since sampling in case-control studies is biased with respect to the disease status i.e., the probability of selection for cases is much higher than that of controls [46, 47], CCW-TMLE is a weighted analysis. The weights were calculated as follows: The total number of BC women who were registered at the center was 1020.As 85% of newly-diagnosed cases were referred to this center [41], over the period of the study, there were 1020/0.85 = 1200 newly diagnosed patients in the province. Of these, 787 patients (with complete information) were entered into our study. Thus the sampling fraction of the case group was 787/1200 and the weight for cases will be 1200/787 = 1.5248. The average population of women over 20 years old in the study period in Fars province was 1,346,630. In this study, 928 women were selected as the control group. Thus, the sampling fraction of the control group is equal to 928/1,346,630 and the weight for the control group will be 1,346,630/928 = 1451.1.

The steps of CCW-TMLE are as follows:

- ***Step 1:*** The case and control weights described above were assigned to cases and controls necessary due to the nature of the case-control study, to simulate a cohort study.
- ***Step 2:*** The weighted conditional distribution of the outcome given exposure and confounders was estimated using super learning.
- ***Step 3:*** The weighted conditional distribution of the exposure given confounders was estimated using super learning.
- ***Step 4:*** A clever covariate, the inverse probability of exposure given confounders in the case group and the negative of the inverse probability of no exposure given confounders in the control group, was calculated.
- ***Step 5:*** The outcome regression model from Step 2 was updated by adding the covariate described in step 4, so that the coefficients of the model do not change.
- ***Step 6:*** The standardized mean outcome (e.g., risk) in the exposed group was calculated by predicting the individual mean outcome, for exposure forced to be 1 for all individuals, and the actual values of confounders, and then averaging them over the individuals from the model fitted in Step 5. Similarly, we calculated the standardized mean outcome (e.g., risk) in the unexposed group by predicting the individual mean outcome, for exposure forced to be 0 for all individuals, and the actual values of confounders, and then averaging them over the individuals from the model fitted in Step 5. Then we derived the RD, RR, and PAF.
- ***Step 7:*** The efficient influence curve (EIC) was used to estimate the standard error and compute Wald-type 95% confidence intervals (CIs) [29, 30, 49].

In our study, PAF measures the proportion of BC (or any health-related outcome) that is attributable to a given exposure or the proportion of all BC cases that would not have occurred if the exposure has been removed [50, 51] and was calculated as follow [52]:

$$ \mathrm{PAF}=\frac{{\mathrm{P}}_{\mathrm{c}}\left( RR-1\right)}{RR} $$

where P_c_ stands for the prevalence of exposure in the case group. To calculate a 95% CI for the PAF, a bootstrap confidence interval was used based on 10,000 bootstrap replicates and reporting the 2.5th and 97.5th percentiles.

### Statistical software

Stata 14.0 (StataCorp LLC, College Station, Texas, USA) and R 3–4-3 software (R Foundation for Statistical Computing, Vienna, Austria) were used to perform the statistical analyses. The super learner packages *glm, step, glm.interaction, randomForest, gam, rpart and glmnet algorithm*s were used. The codes used in the statistical analyses have been provided as a supplementary file for reproducibility (Appendix 1).

## Results

The demographic variables have been described, separately for case and control groups, in Table 1. The mean age in the case and control groups were 49.8 (SD = 0.4) and 49.7 (SD = 0.3) years, respectively (*p* = 0.8). The body mass index in the case group was higher than that in the control group (27.9 vs. 27.3 kg/m^2^; *p* = 0.001).

**Table 1:** Comparison of demographic continuous variables by case-control status (787 cases and 928 controls) in Fars province, Iran, 2009

Characteristic	Cases (*n* = 787)	Controls (*n* = 928)	*P*-value^†^
	Mean (SD)	Mean (SD)	
Age (year)	49.8 (0.36)	49.7 (0.34)	0.820
Height (cm)	156.3 (0.21)	156.6 (0.20)	0.270
Weight (kg)	68.3 (0.42)	66.9 (0.39)	0.010
Body mass index^*^	27.9 (0.16)	27.3 (0.15)	0.001

^*^Weight (kg)/height^2^ (m^2^); ^†^Obtained from independent t-test

As shown in Table 2, there was strong evidence of higher education level among the case group compared with the control (*p* = 0.001). Also, there was strong evidence that the frequency of being employed was higher in the case group than in the control group (*p* = 0.001), while there was no evidence regarding the difference in marital status between the two groups (*p* = 0.400). Moreover, the results of Table 2 support the higher prevalence of being postmenopausal, being older at first pregnancy, being older at first marriage, breastfeeding duration< 60 months, history of OCP use and parity ≤3 among case group than the control group.

**Table 2:** Comparison of categorical variables by case-control status (787 cases and 928 controls) in Fars province, Iran, 2009

Characteristic		Cases (*n* = 787)		Controls (*n* = 928)		*P*-value^*^
		n	%	n	%	
Education level	Illiterate	171	21.7	371	40.0	0.001
	Primary	284	36.1	303	32.6	
	High-school	239	30.4	217	23.4	
	Academic	93	11.8	37	4.0	
Occupation status	Housewife	635	80.7	873	94.1	0.001
	Employed	152	19.3	55	5.9	
Marital status	Married	665	84.5	777	83.7	0.440
	Divorced	11	1.4	8	0.90	
	Widow	111	14.1	143	15.4	
Menopause status	Pre-Menopausal	330	41.9	476	51.3	0.001
	Post- Menopausal	457	58.1	452	48.7	
Age at first pregnancy (year)	< 25	613	77.9	831	89.55	0.001
	≥25	174	22.1	97	10.45	
Age at first marriage (year)	< 20	478	60.7	713	76.8	0.001
	≥20	309	39.3	215	23.2	
Breastfeeding duration (month)	≤60	475	60.4	385	41.5	0.001
	> 60	312	39.6	643	58.5	
OC use	Never	280	35.6	397	42.8	0.002
	Ever	507	64.4	531	57.2	
Parity	≤3	354	45.0	317	34.2	0.001
	> 3	433	55.0	611	65.8	

*Obtained from chi-square test

**Fig. 1:**
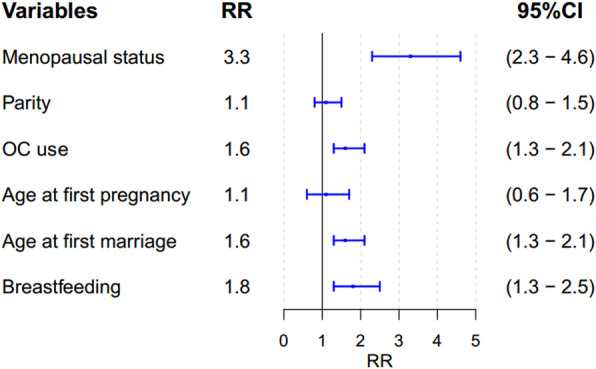
The Risk Ratios (RRs) of Interested Exposure and Breast Cancer

The RR, RD, and PAF, obtained from the TMLE and super learner model, are presented in Table 3. There was no evidence of higher BC risk among women aged ≥25 years at first pregnancy vs. women aged < 25 years (RR = 1.1, 95% CI = (0.6, 1.7)) and there was also no evidence that multiparity (parity> 3) affects the risk of BC (RR = 1.1, 95% CI = (0.8, 1.5)). On the other hand, there was strong evidence supporting a higher risk of BC among postmenopausal women (RR = 3.3, 95% CI = (2.3, 4.6)), women with age at first marriage ≥20 years (RR = 1.6, 95% CI = (1.3, 2.1)), and women with a history of OC use (RR = 1.6, 95% CI = (1.3, 2.1)). Furthermore, the history of lactation ≤60 months had a significant effect on BC (RR = 1.8, 95% CI = (1.3, 2.5)) (Table 3 and Fig. 1). The PAF for menopause status, breastfeeding duration, and history of OC use were 40.3% (95% CI = 39.5, 40.6), 27.3% (95% CI = 23.1, 30.8) and 24.4% (95% CI = 10.5, 35.5), respectively.

**Table 3:** The relationship between reproductive factors and breast cancer risk using TMLE and super learner approach (787 Cases, 928 Controls) in Fars province, Iran, 2009

Reproductive factors	Risk Differences^a^ (95% CI)	Risk ratio (95% CI)	PAF (%) (95% CI)
Parity (> 3)	6.6 (−14.6, 36.7)	1.1 (0.8, 1.5)	3.6 (−8.5, 20.2)
Menopausal status (yes)	69.4 (68.0, 70.0)	3.3 (2.3, 4.6)	40.3 (39.5, 40.6)
Age at first pregnancy (≥25 years)	2.2 (−33.6, 38.1)	1.1 (0.6, 1.7)	1.16 (1.16, 1.17)
Age at first marriage (≥20 years)	37.0 (6.4, 60.1)	1.6 (1.3, 2.1)	14.5 (2.5, 23.5)
Breastfeeding duration (≤60)	45.3 (38.4, 51.1)	1.8 (1.3, 2.5)	27.3 (23.1, 30.8)
OC use (yes)	37.9 (16.8, 56.2)	1.6 (1.3, 2.1)	24.4 (10.5, 35.5)

*CI* confidence interval, *OC* oral contraceptive, *PAF* population attributable fraction. ^a^ per 100,000

## Discussion

Using TMLE and super learner, we examined the causal relationship between reproductive factors and BC in a case-control study. The results showed no evidence of a causal relationship between parity or age at first pregnancy with the risk of BC. However, menopausal status, age at first marriage, duration of breastfeeding, and history of OC use had causal effects on the risk of BC. PAF analysis suggested that menopausal status and breastfeeding duration have the most impact on the risk of BC in our study population. For instance, we found that 27.3% of BC cases in the population can be attributed to a history of breastfeeding duration ≤60 months.

The findings of our study showed no evidence for less or higher risk of BC among multiparous women compared with women with fewer than or equal to three deliveries. A protective role of parity above three has also been reported in a meta-analysis study [15]. according to a study in Nigeria, parity was negatively correlated with the risk of BC [53].. In some other studies, parity has been shown to have a dual effect on BC so that in women under 45 years old parity was considered as a risk factor but in women over 45 years old parity has a protective role [16]. The results of a meta-analysis study have also shown that the risk of BC in women who have not yet given birth is 30% higher than that in women who have given birth, and the risk for every two births is reduced by 16% [54]. The results of the Antoniou et al. study [10] suggested that parity is a protective factor for BC among BRCA1 and BRCA2 notation carriers who were older than 40 years old.

Menopausal status had a strong causal relationship with BC (RR = 3.3, 95% CI: (2.3, 4.6)). Although the results of some studies were in agreement with our results, [13], the others indicated higher risk in premenopausal women in the same age groups [11]. Some of the differences in risk of BC between postmenopausal women and premenopausal women can be explained by differences in estrogen levels and age (if not adjusted) between the two groups [55].

Our study provided no evidence of higher BC risk in women with higher age at first pregnancy. The results of a study in Nigeria failed to show a relationship between age at first live birth and BC [53]. However, several previous studies indicate that low age at the first pregnancy reduces the risk of BC [12, 13, 15, 54, 56] so that the risk of BC has been reported twice among women whose first pregnancy was over 25 years [12]. Being older at first birth has been found to be associated with BC among BRCA2 but not for BRCA1 mutation [10]. The contradiction of our study findings with other studies may be justified by different confounders adjusted for in analysis and statistical methods used; TMLE method, along with the super learner approach, have been identified more efficient for controlling confounding [29, 30, 57].

Although our study suggested that marriage before 20 years old is protective for BC, Ghiasvand et al. failed to demonstrate any relationship between age at first marriage and BC in young women [12]. Our findings are in line with the Kinlen study [58] in which risk of BC in women with age at first marriage and age at first birth 30 years or older was 7 times compared to those with age at first marriage and age at first birth below 20 though he hypothesized that marriage involves the closest contact, pertinent to the infection, leading to an increase in various types of cancer [58, 59].

History of breastfeeding has been shown to be a protective factor for BC with a dose-response relationship, the risk is reduced with an increase in breastfeeding duration [9, 60], confirmed in our study. Generally, two mechanisms have been proposed for the protective effect of breastfeeding, the differentiation of breast tissue and the decrease in the number of ovulation cycles throughout life [61]. the result of a meta-analysis in 2008 demonstrated that only 11 out of the 24 published studies have reported a protective effect of breastfeeding on BC [61].

The history of OC use is considered to be a risk factor for BC as identified in a meta-analysis study by Anothaisintawee et al. [9]. Similarly, the risk in people with a history of OC use in our study was 1.6 times more than that in women without. In a Danish cohort study of 1.8 million women between the ages of 15 and 49, the risk ratio of BC for current and recent OC users was1.2, with more years of consumption, leading to a greater risk [14]. Conversely, some studies have reported any evidence for the effect of OC use on BC [15].

One of the strengths of our case-control study is applying the causal method of weighted TMLE, to identify risk factors of BC, which unlike IPTW and parametric g-formula, is double robust. Using this methodology, we reported risk-based effect measures including, RD and RR as well as the impact measure of PAF. In addition, the super learner method has been employed to estimate the probability of outcome and exposure using a weighted linear combination of different algorithms instead of relying on a single algorithm, to improve the validity and efficiency of the effect estimate.

### Limitations

Causal interpretation requires the measurement of all confounders, some of which may have been ignored in our study so the results should be considered with caution. Similar to all retrospective case-control studies, there was a potential for recall bias. Due to data collection between the September 2005 and December 2008, albeit the risk factors pattern is not expected to change significantly during one or two decades, it is recommended to externally validate the result of our study based on newer data.

## Conclusions

In summary, postmenopausal women, women older at age of marriage, and women with the history of lower breastfeeding duration or OC use are at higher risk of BC. The most important risk and preventive factors were menopausal status and history of breastfeeding duration, respectively. Further studies with a larger sample size and adjustment for a more complete set of confounders, particularly with regard to lifestyle factors, are warranted.

## Supplementary Information

**Additional file 1: Figure s1.** A causal diagram representing the effect of parity on BC in the source population. **Figure s2.** A causal diagram representing the effect of breastfeeding on BC in the source population. **Figure s3.** A causal diagram representing the effect of history of OC usage on BC in the source population. **Figure s4.** A causal diagram representing the effect of menopausal status on BC in the source population. **Figure s5.** A causal diagram representing the effect of age at first pregnancy on BC in the source population. **Figure s6.** A causal diagram representing the effect of age at first marriage on BC in the source population.

**Additional file 2: Appendix 1.** Codes for Case Control Weighted TMLE (CCW-TMLE).

## Data Availability

The data sets used and analyzed during the study are available from the corresponding author on reasonable request.

## Abbreviations

BC: Breast Cancer
CCW-TMLE: Case-Control Weighted Targeted Maximum Likelihood Estimation
CI: Confidence Interval
DAG: Directed Acyclic Graph
IPTW: Inverse Probability of Treatment Weighting
OC: Oral Contraceptive
PAF: Population Attributable Fraction
RR: Risk Ratio
RD: Risk Difference
TMLE: Targeted Maximum Likelihood Estimation
