# Supplementary material for: The causal effect and impact of reproductive factors on breast cancer using super learner and targeted maximum likelihood estimation: a case-control study in Fars Province, Iran
Source: BMC Public Health. 2021 Jun 24;21:1219. doi: 10.1186/s12889-021-11307-5 (PMC8228908; doi:10.1186/s12889-021-11307-5)
Supplement: Supplementary file 1 — Additional file 1: Figure s1. A causal diagram representing the effect of parity on BC in the source population. Figure s2. A causal diagram representing the effect of breastfeeding on BC in the source population. Figure s3. A causal diagram representing the effect of history of OC usage on BC in the source population. Figure s4. A causal diagram representing the effect of menopausal status on BC in the source population. Figure s5. A causal diagram representing the effect of age at first pregnancy on BC in the source population. Figure s6. A causal diagram representing the effect of age at first marriage on BC in the source population. [file 12889_2021_11307_MOESM1_ESM.pdf]

Type of Article: Original Research

Title: The Causal Effect and Impact of Reproductive Factors on Breast Cancer Using Super Learner and Targeted Maximum Likelihood Estimation: A Case-Control Study in Fars Province, Iran

Running Title: Reproductive Factors and Breast Cancer Risk

Authors: Amir Almasi-Hashiani[1,2], Saharnaz Nedjat[3], Reza Ghiasvand[4,5], Saeid Safiri[6,7], Maryam Nazemipour[8,9], Nasrin Mansournia[10], Mohammad Ali Mansournia[11*]

Author's affiliations:

Department of Epidemiology, School of Health, Arak University of Medical Sciences, Arak, Iran

Traditional and Complementary Medicine Research Center, Arak University of Medical Sciences, Arak, Iran

Department of Epidemiology and Biostatistics, School of Public Health, Tehran University of Medical Sciences, Knowledge Utilization Research Center, Tehran University of Medical Science, Tehran, Iran.

Department of Research, Cancer Registry of Norway, Oslo, Norway

Oslo Centre for Biostatistics and Epidemiology, Oslo University Hospital, Oslo, Norway

Aging Research Institute, Tabriz University of Medical Sciences, Tabriz, Iran

Department of Community Medicine, School of Medicine, Tabriz University of Medical Sciences, Tabriz, Iran

Osteoporosis Research Center, Endocrinology and Metabolism Clinical Sciences Institute, Tehran University of Medical Sciences, Tehran, Iran

Psychosocial Health Research Institute, Iran University of Medical Sciences, Tehran, Iran.

Department of Endocrinology, AJA University of Medical Sciences, Tehran, Iran

Department of Epidemiology and Biostatistics, School of Public Health, Tehran University of Medical Sciences, Tehran, Iran

***Corresponding Authors:**

Dr. Mohammad Ali Mansournia,

Department of Epidemiology and Biostatistics, School of Public Health, Tehran University of Medical Sciences, Tehran, Iran, **P.O Box:** 14155-6446, **E-Mail:** mansournia_ma@yahoo.com

Emails:

Amiralmasi2007@gmail.com,

saharnaznedjat@gmail.com,

reza.ghiasvand@medisin.uio.no,

saeidsafiri@gmail.com,

nazemipour@razi.tums.ac.ir

nasrin_sept@yahoo.com

mansournia_ma@yahoo.com,

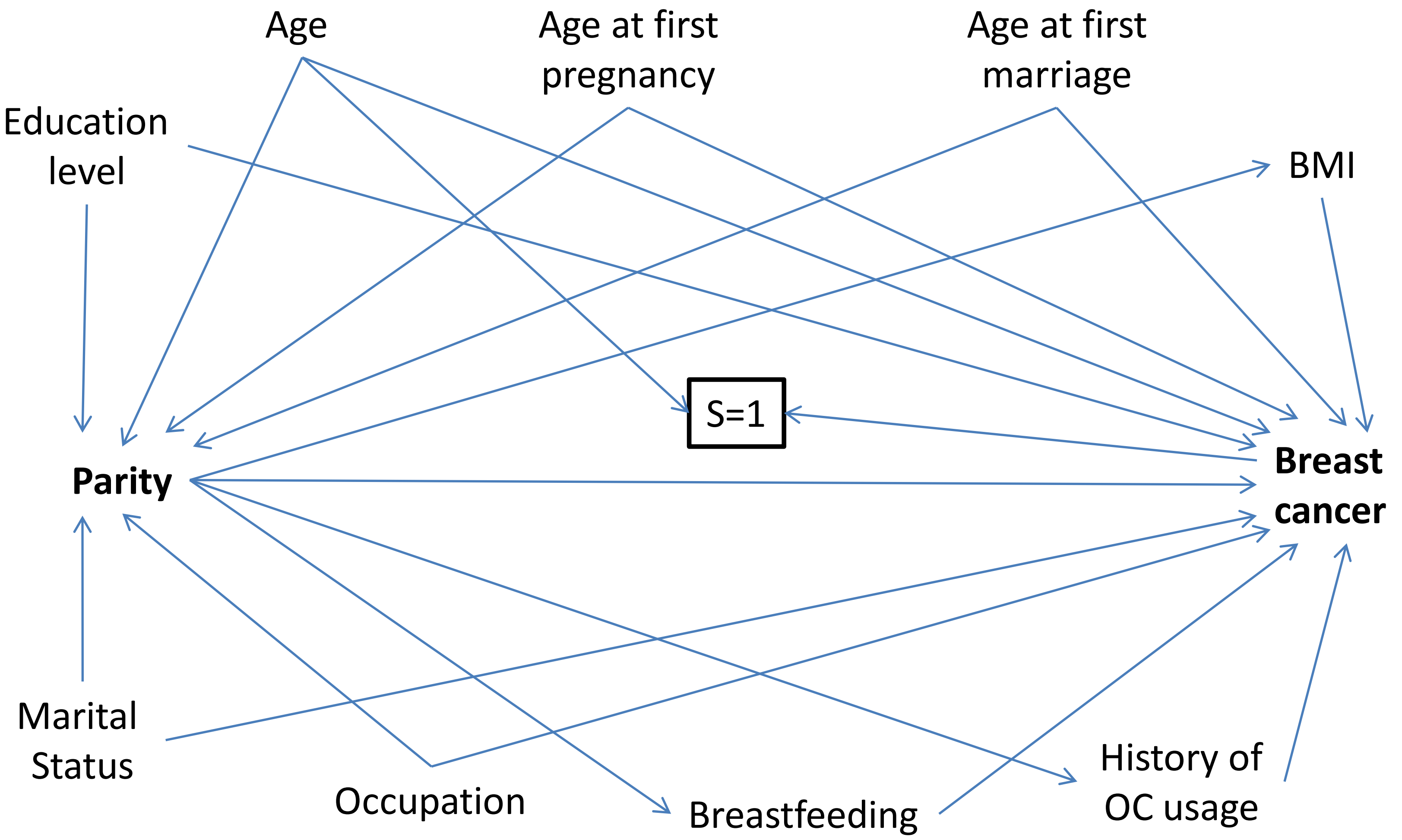

MSS: Age, Age at first pregnancy, Age at first marriage, Occupation, Education and marital status

Figure s1: A causal diagram representing the effect of parity on BC in the source population

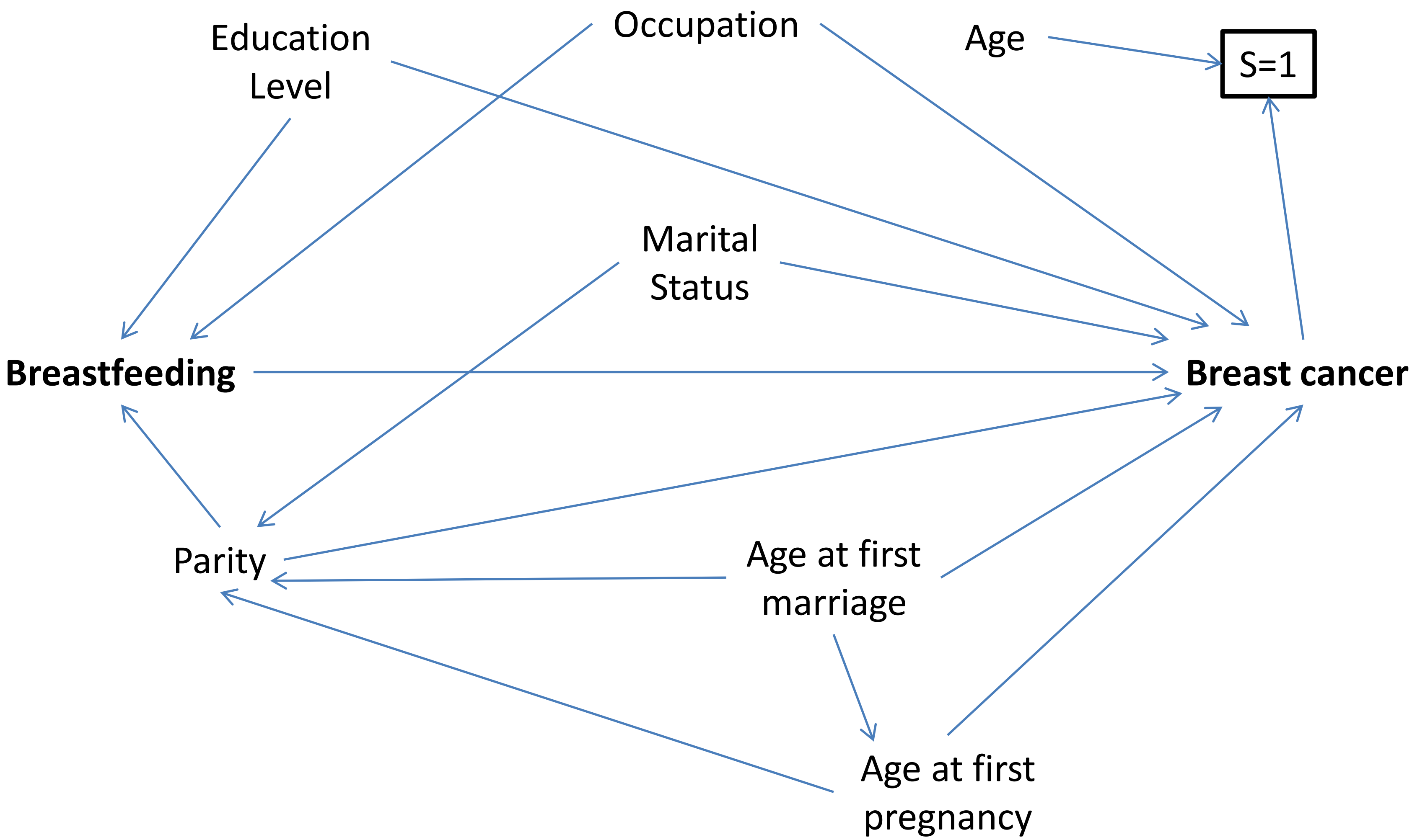

MSS: Occupation, Parity and Education level

Figure s2: A causal diagram representing the effect of breastfeeding on BC in the source population

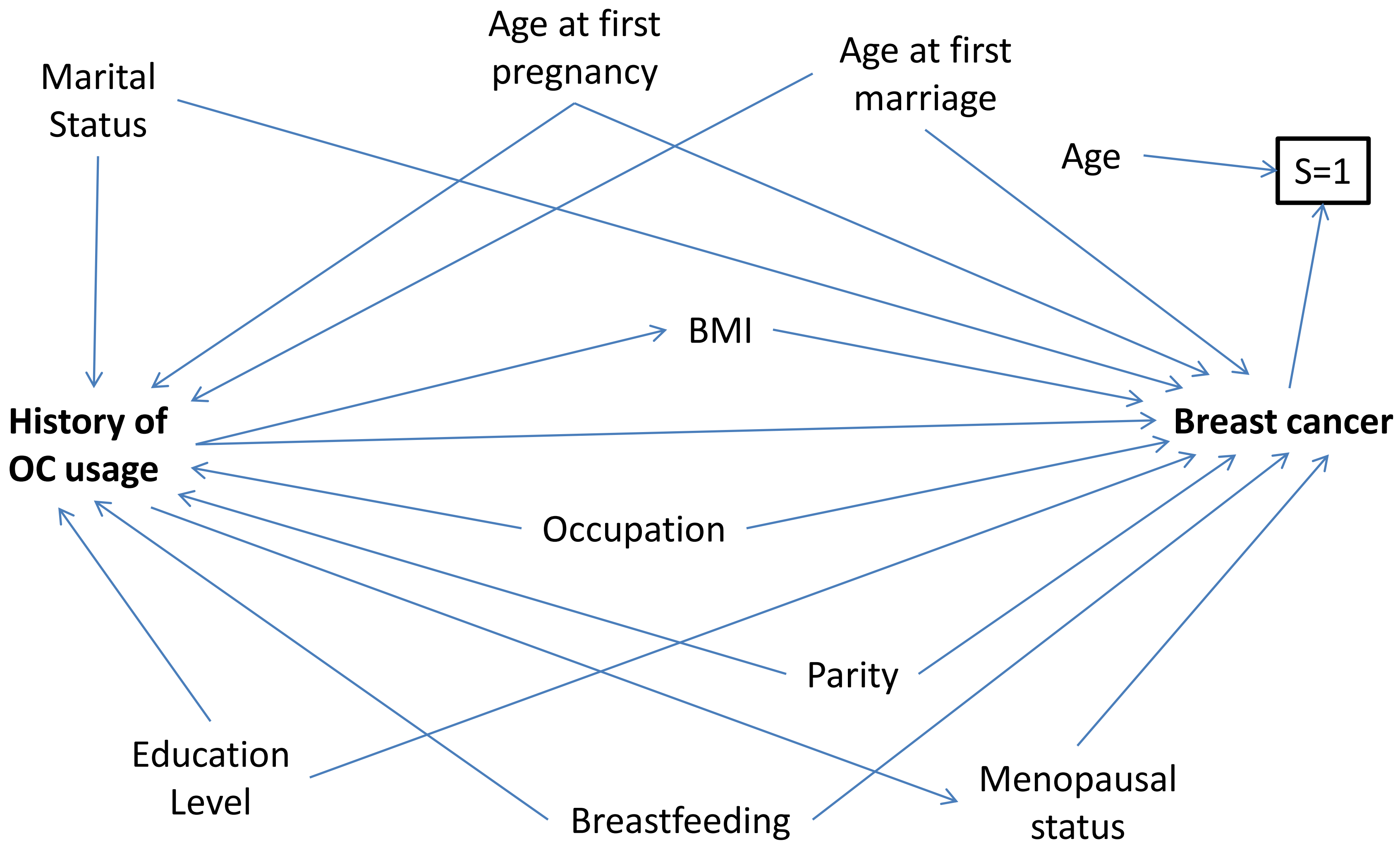

MSS: Age at first pregnancy, Age at first marriage, Parity, Occupation, Marital Status, Education Level and Breastfeeding

Figure s3: A causal diagram representing the effect of history of OC usage on BC in the source population

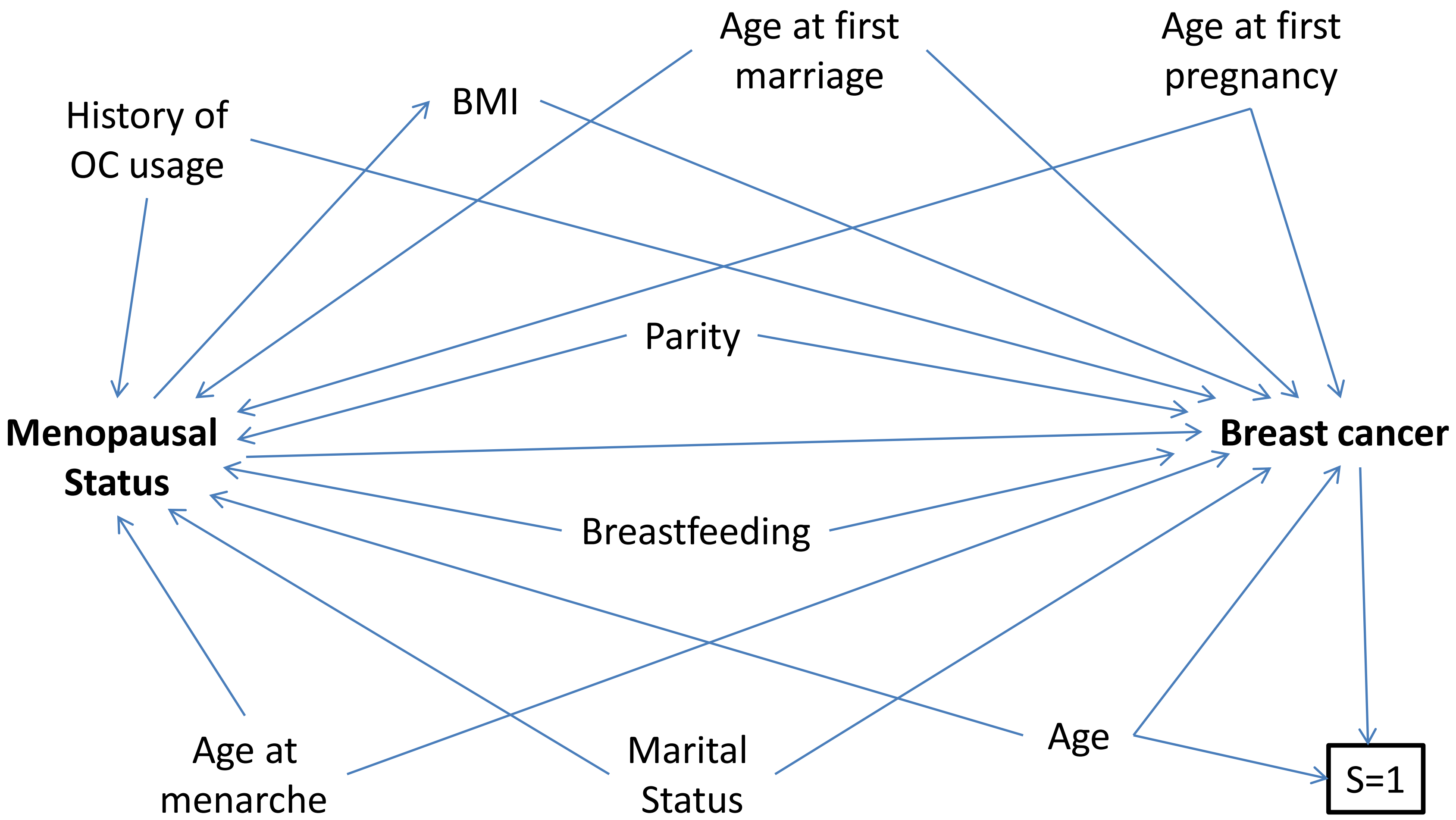

MSS: Parity, Breastfeeding, Age, Age at first marriage, Age at first pregnancy, Age at menarche, Marital Status and History of OC usage

Figure s4: A causal diagram representing the effect of menopausal status on BC in the source population

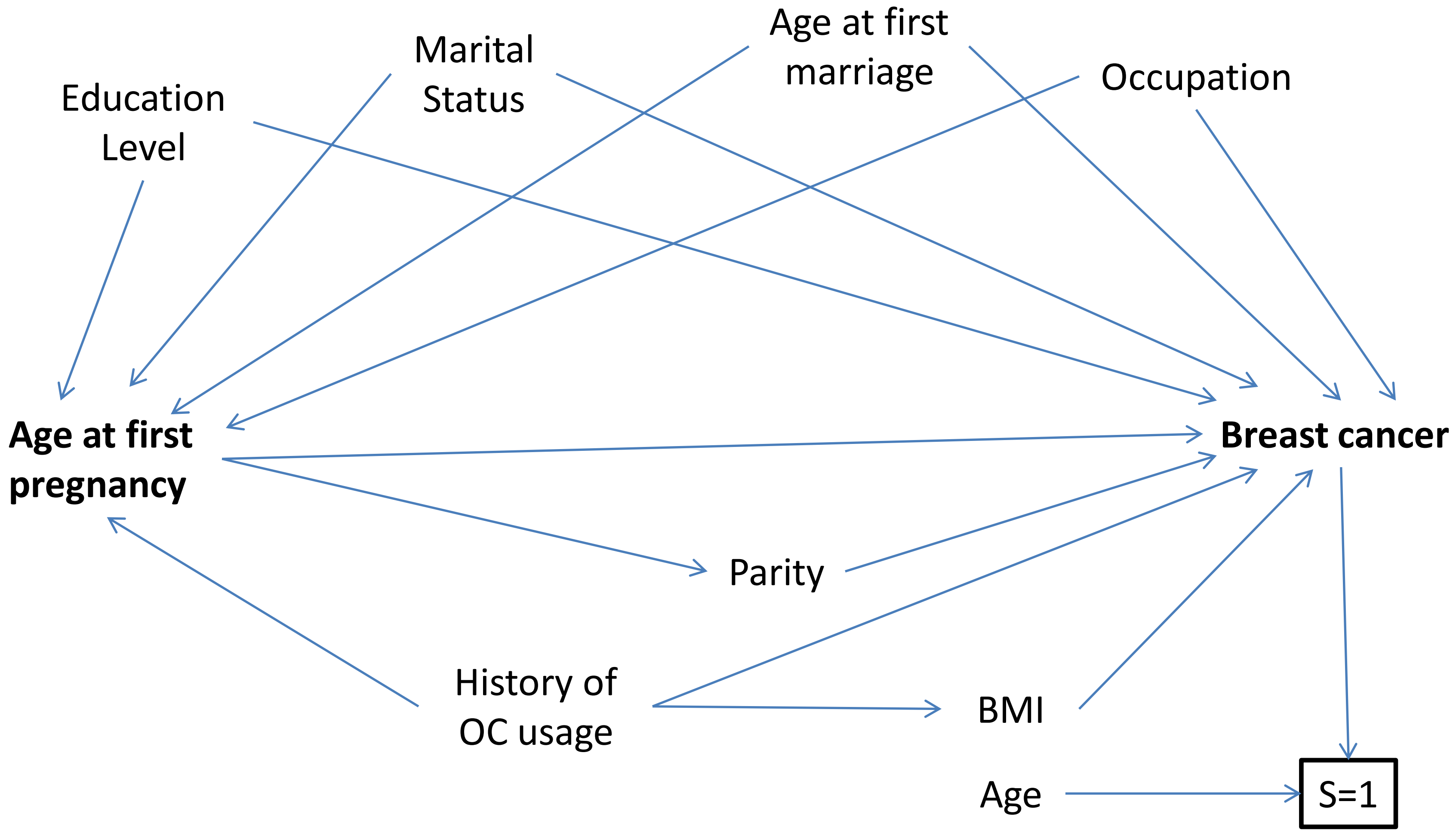

MSS: Age at first marriage, Occupation, Marital Status, Education Level and History of OC usage

Figure s5: A causal diagram representing the effect of age at first pregnancy on BC in the source population

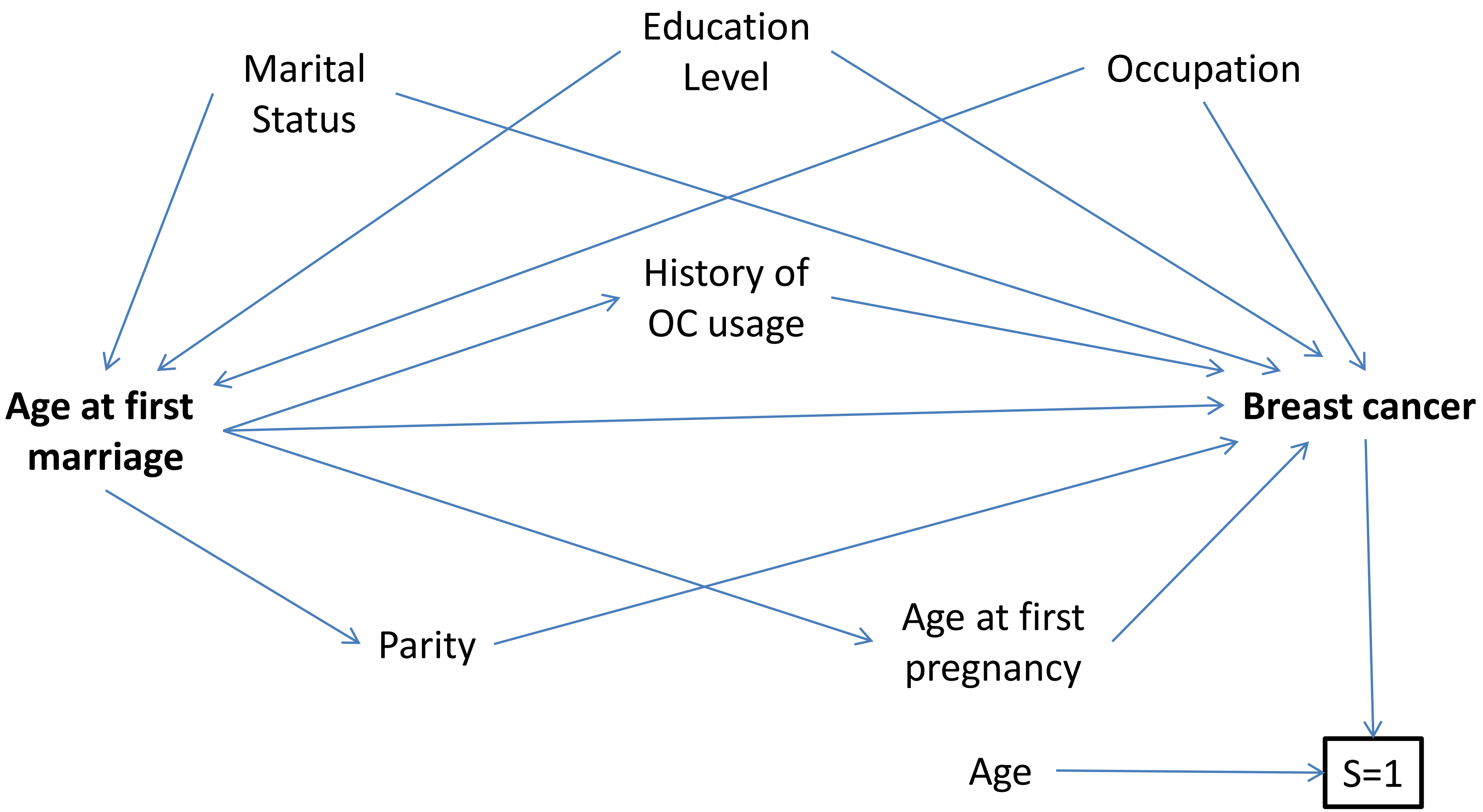

MSS: Occupation, Marital Status, and Education Level

Figure s6: A causal diagram representing the effect of age at first marriage on BC in the source population
