# Supplementary material for: The causal effect and impact of reproductive factors on breast cancer using super learner and targeted maximum likelihood estimation: a case-control study in Fars Province, Iran
Source: BMC Public Health. 2021 Jun 24;21:1219. doi: 10.1186/s12889-021-11307-5 (PMC8228908; doi:10.1186/s12889-021-11307-5)
Supplement: Supplementary file 2 — Additional file 2: Appendix 1. Codes for Case Control Weighted TMLE (CCW-TMLE). [file 12889_2021_11307_MOESM2_ESM.docx]

**Type of Article:** Original Research

**Title**: The Causal Effect and Impact of Reproductive Factors on Breast Cancer Using Super Learner and Targeted Maximum Likelihood Estimation: A Case-Control Study in Fars Province, Iran

**Running Title:** Reproductive Factors and Breast Cancer Risk

**Authors**: Amir Almasi-Hashiani^1,2^, Saharnaz Nedjat^3^, Reza Ghiasvand^4,5^, Saeid Safiri^6,7^, Maryam Nazemipour ^8,9^, Nasrin Mansournia^10^, Mohammad Ali Mansournia^11*^

**Author's affiliations**:

1. Department of Epidemiology, School of Health, Arak University of Medical Sciences, Arak, Iran
2. Traditional and Complementary Medicine Research Center, Arak University of Medical Sciences, Arak, Iran
3. Department of Epidemiology and Biostatistics, School of Public Health, Tehran University of Medical Sciences, Knowledge Utilization Research Center, Tehran University of Medical Science, Tehran, Iran.
4. Department of Research, Cancer Registry of Norway, Oslo, Norway
5. Oslo Centre for Biostatistics and Epidemiology, Oslo University Hospital, Oslo, Norway
6. Aging Research Institute, Tabriz University of Medical Sciences, Tabriz, Iran
7. Department of Community Medicine, School of Medicine, Tabriz University of Medical Sciences, Tabriz, Iran
8. Osteoporosis Research Center, Endocrinology and Metabolism Clinical Sciences Institute, Tehran University of Medical Sciences, Tehran, Iran
9. Psychosocial Health Research Institute, Iran University of Medical Sciences, Tehran, Iran.
10. Department of Endocrinology, AJA University of Medical Sciences, Tehran, Iran
11. Department of Epidemiology and Biostatistics, School of Public Health, Tehran University of Medical Sciences, Tehran, Iran

***Corresponding Authors:**

Dr. Mohammad Ali Mansournia,
Department of Epidemiology and Biostatistics, School of Public Health, Tehran University of Medical Sciences, Tehran, Iran, **P.O Box:** 14155-6446, **E-Mail:** [mansournia_ma@yahoo.com](mailto:mansournia_ma@yahoo.com)

**Emails:**

Amiralmasi2007@gmail.com,

saharnaznedjat@gmail.com,

reza.ghiasvand@medisin.uio.no,

[saeidsafiri@gmail.com](mailto:saeidsafiri@gmail.com),

[nazemipour@razi.tums.ac.ir](mailto:nazemipour@razi.tums.ac.ir)

[nasrin_sept@yahoo.com](mailto:nasrin_sept@yahoo.com)

mansournia_ma@yahoo.com,

**Appendix 1 : Codes for Case Control Weighted TMLE (CCW-TMLE)**

**#####Parity#####**

library(haven)

Thesis_data <- read_dta("Thesis Data.dta")

attach(Thesis_data)

Y=case_control

A= Parity

length(A)

data <- data.frame(id, case_control, Parity, menop_stat, first_pregnancy, first_marriage, menarch2, Breastfeding2, oc_use, cfh2, hrcancers, age, marital_stat, education, oc_duration, family_history, relative_degree, height, weight, bmi, occupation, Wt, A, Y)

library("SuperLearner")

library("ltmle")

library("tmle")

library("rpart")

library("gam")

library("splines")

library("foreach")

library("randomForest")

library("glmnet")

Q.SL.library <- c("SL.glm", "SL.step", "SL.glm.interaction", "SL.randomForest", "SL.gam", "SL.rpart", "SL.glmnet")

g.SL.library <- c("SL.glm", "SL.step", "SL.glm.interaction", "SL.randomForest", "SL.gam", "SL.rpart", "SL.glmnet")

Results1 <- ltmle(

data = data,

Anodes="A",

Lnodes=NULL,

Ynodes="Y",

Qform=c(Y="Q.kplus1 ~ first_pregnancy + first_marriage + age + marital_stat + education + occupation + A"),

gform="A ~ first_pregnancy + first_marriage + age + marital_stat + education + occupation",

abar=list(1, 0),

SL.library = list(Q = Q.SL.library, g = g.SL.library),

variance.method = "ic",

gcomp = FALSE,

iptw.only = FALSE,

observation.weights = as.vector(Wt),

id = NULL

)

print(summary(Results1, estimator="tmle"))

print(Results1$fit)

print(Results1$fit$g$A[, "Coef"])

print(Results1$fit$Q$Y[, "Coef"])

**#####Menopausal Status#####**

library(haven)

Thesis_data <- read_dta("Thesis Data.dta")

attach(Thesis_data)

Y=case_control

A= menop_stat

length(A)

data <- data.frame(id, case_control, Parity, menop_stat, first_pregnancy, first_marriage, menarch2, Breastfeding2, oc_use, cfh2, hrcancers, age, marital_stat, education, oc_duration, family_history, relative_degree, height, weight, bmi, occupation, Wt, A, Y)

library("SuperLearner")

library("ltmle")

library("tmle")

library("rpart")

library("gam")

library("splines")

library("foreach")

library("randomForest")

library("glmnet")

Q.SL.library <- c("SL.glm", "SL.step", "SL.glm.interaction", "SL.randomForest", "SL.gam", "SL.rpart", "SL.glmnet")

g.SL.library <- c("SL.glm", "SL.step", "SL.glm.interaction", "SL.randomForest", "SL.gam", "SL.rpart", "SL.glmnet")

Results2 <- ltmle(

data = data,

Anodes="A",

Lnodes=NULL,

Ynodes="Y",

Qform=c(Y="Q.kplus1 ~ Parity + first_pregnancy + first_marriage + menarch2 + Breastfeding2 + oc_use + age + marital_stat + A"),

gform="A ~ Parity + first_pregnancy + first_marriage + menarch2 + Breastfeding2 + oc_use + age + marital_stat",

abar=list(1, 0),

SL.library = list(Q = Q.SL.library, g = g.SL.library),

variance.method = "ic",

gcomp = FALSE,

iptw.only = FALSE,

observation.weights = as.vector(Wt),

id = NULL

)

print(summary(Results2, estimator="tmle"))

print(Results2$fit)

print(Results2$fit$g$A[, "Coef"])

print(Results2$fit$Q$Y[, "Coef"])

**#####Age at First Pregnancy#####**

library(haven)

Thesis_data <- read_dta("Thesis Data.dta")

attach(Thesis_data)

Y=case_control

A= first_pregnancy

length(A)

data <- data.frame(id, case_control, Parity, menop_stat, first_pregnancy, first_marriage, menarch2, Breastfeding2, oc_use, cfh2, hrcancers, age, marital_stat, education, oc_duration, family_history, relative_degree, height, weight, bmi, occupation, Wt, A, Y)

library("SuperLearner")

library("ltmle")

library("tmle")

library("rpart")

library("gam")

library("splines")

library("foreach")

library("randomForest")

library("glmnet")

Q.SL.library <- c("SL.glm", "SL.step", "SL.glm.interaction", "SL.randomForest", "SL.gam", "SL.rpart", "SL.glmnet")

g.SL.library <- c("SL.glm", "SL.step", "SL.glm.interaction", "SL.randomForest", "SL.gam", "SL.rpart", "SL.glmnet")

Results3 <- ltmle(

data = data,

Anodes="A",

Lnodes=NULL,

Ynodes="Y",

Qform=c(Y="Q.kplus1 ~ first_marriage + age + oc_use + marital_stat + education + occupation"),

gform="A ~ first_marriage + age + oc_use + marital_stat + education + occupation",

abar=list(1, 0),

SL.library = list(Q = Q.SL.library, g = g.SL.library),

variance.method = "ic",

gcomp = FALSE,

iptw.only = FALSE,

observation.weights = as.vector(Wt),

id = NULL

)

print(summary(Results3, estimator="tmle"))

print(Results3$fit)

print(Results3$fit$g$A[, "Coef"])

print(Results3$fit$Q$Y[, "Coef"])

**#####Age at First Marriage#####**

library(haven)

Thesis_data <- read_dta("Thesis Data.dta")

attach(Thesis_data)

Y= case_control

A= first_marriage

length(A)

data <- data.frame(id, case_control, Parity, menop_stat, first_pregnancy, first_marriage, menarch2, Breastfeding2, oc_use, cfh2, hrcancers, age, marital_stat, education, oc_duration, family_history, relative_degree, height, weight, bmi, occupation, Wt, A, Y)

library("SuperLearner")

library("ltmle")

library("tmle")

library("rpart")

library("gam")

library("splines")

library("foreach")

library("randomForest")

library("glmnet")

Q.SL.library <- c("SL.glm", "SL.step", "SL.glm.interaction", "SL.randomForest", "SL.gam", "SL.rpart", "SL.glmnet")

g.SL.library <- c("SL.glm", "SL.step", "SL.glm.interaction", "SL.randomForest", "SL.gam", "SL.rpart", "SL.glmnet")

Results4 <- ltmle(

data = data,

Anodes="A",

Lnodes=NULL,

Ynodes="Y",

Qform=c(Y="Q.kplus1 ~ marital_stat + age + education + occupation + A"),

gform="A ~ marital_stat + age + education + occupation",

abar=list(1, 0),

SL.library = list(Q = Q.SL.library, g = g.SL.library),

variance.method = "ic",

gcomp = FALSE,

iptw.only = FALSE,

observation.weights = as.vector(Wt),

id = NULL

)

print(summary(Results4, estimator="tmle"))

print(Results4$fit)

print(Results4$fit$g$A[, "Coef"])

print(Results4$fit$Q$Y[, "Coef"])

**#####Breastfeeding#####**

library(haven)

Thesis_data <- read_dta("Thesis Data.dta")

attach(Thesis_data)

Y= case_control

A= Breastfeding2

length(A)

data <- data.frame(id, case_control, Parity, menop_stat, first_pregnancy, first_marriage, menarch2, Breastfeding2, oc_use, cfh2, hrcancers, age, marital_stat, education, oc_duration, family_history, relative_degree, height, weight, bmi, occupation, Wt, A, Y)

library("SuperLearner")

library("ltmle")

library("tmle")

library("rpart")

library("gam")

library("splines")

library("foreach")

library("randomForest")

library("glmnet")

Q.SL.library <- c("SL.glm", "SL.step", "SL.glm.interaction", "SL.randomForest", "SL.gam", "SL.rpart", "SL.glmnet")

g.SL.library <- c("SL.glm", "SL.step", "SL.glm.interaction", "SL.randomForest", "SL.gam", "SL.rpart", "SL.glmnet")

Results5 <- ltmle(

data = data,

Anodes="A",

Lnodes=NULL,

Ynodes="Y",

Qform=c(Y="Q.kplus1 ~ Parity + age + education + occupation + A"),

gform="A ~ Parity + age + education + occupation",

abar=list(1, 0),

SL.library = list(Q = Q.SL.library, g = g.SL.library),

variance.method = "ic",

gcomp = FALSE,

iptw.only = FALSE,

observation.weights = as.vector(Wt),

id = NULL

)

print(summary(Results5, estimator="tmle"))

print(Results5$fit)

print(Results5$fit$g$A[, "Coef"])

print(Results5$fit$Q$Y[, "Coef"])

**#####OCP Use#####**

library(haven)

Thesis_data <- read_dta("Thesis Data.dta")

attach(Thesis_data)

Y= case_control

A= oc_use

length(A)

data <- data.frame(id, case_control, Parity, menop_stat, first_pregnancy, first_marriage, menarch2, Breastfeding2, oc_use, cfh2, hrcancers, age, marital_stat, education, oc_duration, family_history, relative_degree, height, weight, bmi, occupation, Wt, A, Y)

library("SuperLearner")

library("ltmle")

library("tmle")

library("rpart")

library("gam")

library("splines")

library("foreach")

library("randomForest")

library("glmnet")

Q.SL.library <- c("SL.glm", "SL.step", "SL.glm.interaction", "SL.randomForest", "SL.gam", "SL.rpart", "SL.glmnet")

g.SL.library <- c("SL.glm", "SL.step", "SL.glm.interaction", "SL.randomForest", "SL.gam", "SL.rpart", "SL.glmnet")

Results6 <- ltmle(

data = data,

Anodes="A",

Lnodes=NULL,

Ynodes="Y",

Qform=c(Y="Q.kplus1 ~ Parity + age + first_pregnancy + first_marriage + Breastfeding2 + marital_stat + education + occupation + A"),

gform="A ~ Parity + age + first_pregnancy + first_marriage + Breastfeding2 + marital_stat + education + occupation",

abar=list(1, 0),

SL.library = list(Q = Q.SL.library, g = g.SL.library),

variance.method = "ic",

gcomp = FALSE,

iptw.only = FALSE,

observation.weights = as.vector(Wt),

id = NULL

)

print(summary(Results6, estimator="tmle"))

print(Results6$fit)

print(Results6$fit$g$A[, "Coef"])

print(Results6$fit$Q$Y[, "Coef"])

**Codes for TMLE:** **Excess Risk and Population Attributable Fraction (PAF)**

**####Parity####**

library(haven)

Thesis_Data <- read_dta("C:/Documents and Settings/toshiba/Desktop/Thesis/Thesis Report-7.5.97/Thesis Dta and Codes/Thesis Data.dta")

attach(Thesis_Data)

Y=case_control

A= Parity

data <- data.frame(id, case_control, Parity, menop_stat, first_pregnancy, first_marriage, menarch2, Breastfeding2, oc_use, cfh2, hrcancers, age, marital_stat, education, oc_duration, family_history, relative_degree, height, weight, bmi, occupation, Wt, A, Y)

# Percentage exposed among cases

pec <- 0.5502 # Update it based on your data

# Specify number of cases and number of controls (Based on your data)

nCa<- 787

nCo<- 928

n<- nCa + nCo

J<- nCo/nCa # ratio of number controls to cases

q<- mean(Y)

q

library(ltmle)

library("SuperLearner")

library("ltmle")

library("tmle")

library("rpart")

library("gam")

library("splines")

library("foreach")

library("randomForest")

library("glmnet")

Q.SL.library <- c("SL.glm", "SL.step", "SL.glm.interaction", "SL.randomForest", "SL.gam", "SL.rpart", "SL.glmnet")

g.SL.library <- c("SL.glm", "SL.step", "SL.glm.interaction", "SL.randomForest", "SL.gam", "SL.rpart", "SL.glmnet")

R <- ltmle(

data = data,

Anodes="A",

Lnodes = NULL,

Cnodes = NULL,

Ynodes="Y",

abar=list(1, 0),

Qform=c(Y="Q.kplus1 ~ first_pregnancy + first_marriage + age + marital_stat + education + occupation + A"),

gform="A ~ first_pregnancy + first_marriage + age + marital_stat + education + occupation",

SL.library = list(Q = Q.SL.library, g = g.SL.library),

variance.method = "ic",

gcomp = FALSE,

iptw.only = FALSE,

observation.weights = as.vector(Wt),

id = NULL

)

print(summary(R, estimator="tmle"))

# Extract information from the ltmle R object

Q <- as.data.frame(R$Qstar)

EY0 <- mean(Q[,2]);EY0

EY1 <- mean(Q[,1]);EY1

ATE <- mean(Q[,1]-Q[,2]);ATE

RR <- EY1/EY0; RR

################################################################################

# Excess Risk (Boostraping for 95%CI)

################################################################################

ER <- mean(1 - Q[,2]/Q[,1]);ER

library(boot)

ER.w= function(Q,indices)

{

dat=Q[indices,]

(1 - (dat$V2)/(dat$V1))

}

# Can get original estimate, by plugging in indices 1:n

ER.w(Q,indices=1:nrow(Q))

# Draw 10000 bootstrap sample estimates

boot.out=boot(Q,ER.w,10000)

# Compute confidence intervals using percentile method

boot.ci(boot.out,type="perc",conf=0.95)

ER

#############################################################

# Population Attributable Fraction (Boostraping for 95%CI)

#############################################################

ATF <- mean(pec * (1 - Q[,2]/Q[,1])); ATF

library(boot)

ATF.w= function(Q,indices)

{

dat=Q[indices,]

pec * (1 - (dat$V2)/(dat$V1))

}

# Can get original estimate, by plugging in indices 1:n

ATF.w(Q,indices=1:nrow(Q))

# Draw 10000 bootstrap sample estimates

boot.out=boot(Q,ATF.w,10000)

# Compute confidence intervals using percentile method

boot.ci(boot.out,type="perc",conf=0.95)

ATF

**####Menopausal Status####**

library(haven)

Thesis_Data <- read_dta("C:/Documents and Settings/toshiba/Desktop/Thesis/Thesis Report-7.5.97/Thesis Dta and Codes/Thesis Data.dta")

attach(Thesis_Data)

Y=case_control

A= menop_stat

data <- data.frame(id, case_control, Parity, menop_stat, first_pregnancy, first_marriage, menarch2, Breastfeding2, oc_use, cfh2, hrcancers, age, marital_stat, education, oc_duration, family_history, relative_degree, height, weight, bmi, occupation, Wt, A, Y)

# Percentage exposed among cases

pec <- 0.5807 # Update it based on your data

# Specify number of cases and number of controls (Based on your data)

nCa<- 787

nCo<- 928

n<- nCa + nCo

J<- nCo/nCa # ratio of number controls to cases

q<- mean(Y)

q

library(ltmle)

library("SuperLearner")

library("ltmle")

library("tmle")

library("rpart")

library("gam")

library("splines")

library("foreach")

library("randomForest")

library("glmnet")

Q.SL.library <- c("SL.glm", "SL.step", "SL.glm.interaction", "SL.randomForest", "SL.gam", "SL.rpart", "SL.glmnet")

g.SL.library <- c("SL.glm", "SL.step", "SL.glm.interaction", "SL.randomForest", "SL.gam", "SL.rpart", "SL.glmnet")

R <- ltmle(

data = data,

Anodes="A",

Lnodes = NULL,

Cnodes = NULL,

Ynodes="Y",

abar=list(1, 0),

Qform=c(Y="Q.kplus1 ~ Parity + first_pregnancy + first_marriage + menarch2 + Breastfeding2 + oc_use + age + marital_stat + A"),

gform="A ~ Parity + first_pregnancy + first_marriage + menarch2 + Breastfeding2 + oc_use + age + marital_stat",

SL.library = list(Q = Q.SL.library, g = g.SL.library),

variance.method = "ic",

gcomp = FALSE,

iptw.only = FALSE,

observation.weights = as.vector(Wt),

id = NULL

)

print(summary(R, estimator="tmle"))

# Extract information from the ltmle R object

Q <- as.data.frame(R$Qstar)

EY0 <- mean(Q[,2]);EY0

EY1 <- mean(Q[,1]);EY1

ATE <- mean(Q[,1]-Q[,2]);ATE

RR <- EY1/EY0; RR

#################################################################################

# Excess Risk (Boostraping for 95%CI)

################################################################################

ER <- mean(1 - Q[,2]/Q[,1]);ER

library(boot)

ER.w= function(Q,indices)

{

dat=Q[indices,]

(1 - (dat$V2)/(dat$V1))

}

# Can get original estimate, by plugging in indices 1:n

ER.w(Q,indices=1:nrow(Q))

# Draw 10000 bootstrap sample estimates

boot.out=boot(Q,ER.w,10000)

# Compute confidence intervals using percentile method

boot.ci(boot.out,type="perc",conf=0.95)

ER

#############################################################

# Population Attributable Fraction (Boostraping for 95%CI)

#############################################################

ATF <- mean(pec * (1 - Q[,2]/Q[,1])); ATF

library(boot)

ATF.w= function(Q,indices)

{

dat=Q[indices,]

pec * (1 - (dat$V2)/(dat$V1))

}

# Can get original estimate, by plugging in indices 1:n

ATF.w(Q,indices=1:nrow(Q))

# Draw 10000 bootstrap sample estimates

boot.out=boot(Q,ATF.w,10000)

# Compute confidence intervals using percentile method

boot.ci(boot.out,type="perc",conf=0.95)

ATF

**####Age at First Pregnancy####**

library(haven)

Thesis_Data <- read_dta("C:/Documents and Settings/toshiba/Desktop/Thesis/Thesis Report-7.5.97/Thesis Dta and Codes/Thesis Data.dta")

attach(Thesis_Data)

Y=case_control

A= first_pregnancy

data <- data.frame(id, case_control, Parity, menop_stat, first_pregnancy, first_marriage, menarch2, Breastfeding2, oc_use, cfh2, hrcancers, age, marital_stat, education, oc_duration, family_history, relative_degree, height, weight, bmi, occupation, Wt, A, Y)

# Percentage exposed among cases

pec <- 0.2211 # Update it based on your data

# Specify number of cases and number of controls (Based on your data)

nCa<- 787

nCo<- 928

n<- nCa + nCo

J<- nCo/nCa # ratio of number controls to cases

q<- mean(Y)

q

library("ltmle")

library("SuperLearner")

library("ltmle")

library("tmle")

library("rpart")

library("gam")

library("splines")

library("foreach")

library("randomForest")

library("glmnet")

Q.SL.library <- c("SL.glm", "SL.step", "SL.glm.interaction", "SL.randomForest", "SL.gam", "SL.rpart", "SL.glmnet")

g.SL.library <- c("SL.glm", "SL.step", "SL.glm.interaction", "SL.randomForest", "SL.gam", "SL.rpart", "SL.glmnet")

R <- ltmle(

data = data,

Anodes="A",

Lnodes = NULL,

Cnodes = NULL,

Ynodes="Y",

abar=list(1, 0),

Qform=c(Y="Q.kplus1 ~ first_marriage + age + oc_use + marital_stat + education + occupation"),

gform="A ~ first_marriage + age + oc_use + marital_stat + education + occupation",

SL.library = list(Q = Q.SL.library, g = g.SL.library),

variance.method = "ic",

gcomp = FALSE,

iptw.only = FALSE,

observation.weights = as.vector(Wt),

id = NULL

)

print(summary(R, estimator="tmle"))

# Extract information from the ltmle R object

Q <- as.data.frame(R$Qstar)

EY0 <- mean(Q[,2]);EY0

EY1 <- mean(Q[,1]);EY1

ATE <- mean(Q[,1]-Q[,2]);ATE

RR <- EY1/EY0; RR

#################################################################################

# Excess Risk (Boostraping for 95%CI)

################################################################################

ER <- mean(1 - Q[,2]/Q[,1]);ER

library(boot)

ER.w= function(Q,indices)

{

dat=Q[indices,]

(1 - (dat$V2)/(dat$V1))

}

# Can get original estimate, by plugging in indices 1:n

ER.w(Q,indices=1:nrow(Q))

# Draw 10000 bootstrap sample estimates

boot.out=boot(Q,ER.w,10000)

# Compute confidence intervals using percentile method

boot.ci(boot.out,type="perc",conf=0.95)

ER

#############################################################

# Population Attributable Fraction (Boostraping for 95%CI)

#############################################################

ATF <- mean(pec * (1 - Q[,2]/Q[,1])); ATF

library(boot)

ATF.w= function(Q,indices)

{

dat=Q[indices,]

pec * (1 - (dat$V2)/(dat$V1))

}

# Can get original estimate, by plugging in indices 1:n

ATF.w(Q,indices=1:nrow(Q))

# Draw 10000 bootstrap sample estimates

boot.out=boot(Q,ATF.w,10000)

# Compute confidence intervals using percentile method

boot.ci(boot.out,type="perc",conf=0.95)

ATF

**####Age at First Marriage####**

library(haven)

Thesis_Data <- read_dta("C:/Documents and Settings/toshiba/Desktop/Thesis/Thesis Report-7.5.97/Thesis Dta and Codes/Thesis Data.dta")

attach(Thesis_Data)

Y=case_control

A= first_marriage

data <- data.frame(id, case_control, Parity, menop_stat, first_pregnancy, first_marriage, menarch2, Breastfeding2, oc_use, cfh2, hrcancers, age, marital_stat, education, oc_duration, family_history, relative_degree, height, weight, bmi, occupation, Wt, A, Y)

# Percentage exposed among cases

pec <- 0.3926 # Update it based on your data

# Specify number of cases and number of controls (Based on your data)

nCa<- 787

nCo<- 928

n<- nCa + nCo

J<- nCo/nCa # ratio of number controls to cases

q<- mean(Y)

q

library("ltmle")

library("SuperLearner")

library("ltmle")

library("tmle")

library("rpart")

library("gam")

library("splines")

library("foreach")

library("randomForest")

library("glmnet")

Q.SL.library <- c("SL.glm", "SL.step", "SL.glm.interaction", "SL.randomForest", "SL.gam", "SL.rpart", "SL.glmnet")

g.SL.library <- c("SL.glm", "SL.step", "SL.glm.interaction", "SL.randomForest", "SL.gam", "SL.rpart", "SL.glmnet")

R <- ltmle(

data = data,

Anodes="A",

Lnodes = NULL,

Cnodes = NULL,

Ynodes="Y",

abar=list(1, 0),

Qform=c(Y="Q.kplus1 ~ marital_stat + age + education + occupation + A"),

gform="A ~ marital_stat + age + education + occupation",

SL.library = list(Q = Q.SL.library, g = g.SL.library),

variance.method = "ic",

gcomp = FALSE,

iptw.only = FALSE,

observation.weights = as.vector(Wt),

id = NULL

)

print(summary(R, estimator="tmle"))

# Extract information from the ltmle R object

Q <- as.data.frame(R$Qstar)

EY0 <- mean(Q[,2]);EY0

EY1 <- mean(Q[,1]);EY1

ATE <- mean(Q[,1]-Q[,2]);ATE

RR <- EY1/EY0; RR

#################################################################################

# Excess Risk (Boostraping for 95%CI)

################################################################################

ER <- mean(1 - Q[,2]/Q[,1]);ER

library(boot)

ER.w= function(Q,indices)

{

dat=Q[indices,]

(1 - (dat$V2)/(dat$V1))

}

# Can get original estimate, by plugging in indices 1:n

ER.w(Q,indices=1:nrow(Q))

# Draw 10000 bootstrap sample estimates

boot.out=boot(Q,ER.w,10000)

# Compute confidence intervals using percentile method

boot.ci(boot.out,type="perc",conf=0.95)

ER

#############################################################

# Population Attributable Fraction (Boostraping for 95%CI)

#############################################################

ATF <- mean(pec * (1 - Q[,2]/Q[,1])); ATF

library(boot)

ATF.w= function(Q,indices)

{

dat=Q[indices,]

pec * (1 - (dat$V2)/(dat$V1))

}

# Can get original estimate, by plugging in indices 1:n

ATF.w(Q,indices=1:nrow(Q))

# Draw 10000 bootstrap sample estimates

boot.out=boot(Q,ATF.w,10000)

# Compute confidence intervals using percentile method

boot.ci(boot.out,type="perc",conf=0.95)

ATF

**####Breastfeeding####**

library(haven)

Thesis_Data <- read_dta("C:/Documents and Settings/toshiba/Desktop/Thesis/Thesis Report-7.5.97/Thesis Dta and Codes/Thesis Data.dta")

attach(Thesis_Data)

Y=case_control

A= Breastfeding2

data <- data.frame(id, case_control, Parity, menop_stat, first_pregnancy, first_marriage, menarch2, Breastfeding2, oc_use, cfh2, hrcancers, age, marital_stat, education, oc_duration, family_history, relative_degree, height, weight, bmi, occupation, Wt, A, Y)

# Percentage exposed among cases

pec <- 0.6036 # Update it based on your data

# Specify number of cases and number of controls (Based on your data)

nCa<- 787

nCo<- 928

n<- nCa + nCo

J<- nCo/nCa # ratio of number controls to cases

q<- mean(Y)

q

library("ltmle")

library("SuperLearner")

library("ltmle")

library("tmle")

library("rpart")

library("gam")

library("splines")

library("foreach")

library("randomForest")

library("glmnet")

Q.SL.library <- c("SL.glm", "SL.step", "SL.glm.interaction", "SL.randomForest", "SL.gam", "SL.rpart", "SL.glmnet")

g.SL.library <- c("SL.glm", "SL.step", "SL.glm.interaction", "SL.randomForest", "SL.gam", "SL.rpart", "SL.glmnet")

R <- ltmle(

data = data,

Anodes="A",

Lnodes = NULL,

Cnodes = NULL,

Ynodes="Y",

abar=list(1, 0),

Qform=c(Y="Q.kplus1 ~ Parity + age + education + occupation + A"),

gform="A ~ Parity + age + education + occupation",

SL.library = list(Q = Q.SL.library, g = g.SL.library),

variance.method = "ic",

gcomp = FALSE,

iptw.only = FALSE,

observation.weights = as.vector(Wt),

id = NULL

)

print(summary(R, estimator="tmle"))

# Extract information from the ltmle R object

Q <- as.data.frame(R$Qstar)

EY0 <- mean(Q[,2]);EY0

EY1 <- mean(Q[,1]);EY1

ATE <- mean(Q[,1]-Q[,2]);ATE

RR <- EY1/EY0; RR

#################################################################################

# Excess Risk (Boostraping for 95%CI)

################################################################################

ER <- mean(1 - Q[,2]/Q[,1]);ER

library(boot)

ER.w= function(Q,indices)

{

dat=Q[indices,]

(1 - (dat$V2)/(dat$V1))

}

# Can get original estimate, by plugging in indices 1:n

ER.w(Q,indices=1:nrow(Q))

# Draw 10000 bootstrap sample estimates

boot.out=boot(Q,ER.w,10000)

# Compute confidence intervals using percentile method

boot.ci(boot.out,type="perc",conf=0.95)

ER

#############################################################

# Population Attributable Fraction (Boostraping for 95%CI)

#############################################################

ATF <- mean(pec * (1 - Q[,2]/Q[,1])); ATF

library(boot)

ATF.w= function(Q,indices)

{

dat=Q[indices,]

pec * (1 - (dat$V2)/(dat$V1))

}

# Can get original estimate, by plugging in indices 1:n

ATF.w(Q,indices=1:nrow(Q))

# Draw 10000 bootstrap sample estimates

boot.out=boot(Q,ATF.w,10000)

# Compute confidence intervals using percentile method

boot.ci(boot.out,type="perc",conf=0.95)

ATF

**####OCP Use####**

library(haven)

Thesis_Data <- read_dta("C:/Documents and Settings/toshiba/Desktop/Thesis/Thesis Report-7.5.97/Thesis Dta and Codes/Thesis Data.dta")

attach(Thesis_Data)

Y=case_control

A= oc_use

data <- data.frame(id, case_control, Parity, menop_stat, first_pregnancy, first_marriage, menarch2, Breastfeding2, oc_use, cfh2, hrcancers, age, marital_stat, education, oc_duration, family_history, relative_degree, height, weight, bmi, occupation, Wt, A, Y)

# Percentage exposed among cases

pec <- 0.6442 # Update it based on your data

# Specify number of cases and number of controls (Based on your data)

nCa<- 787

nCo<- 928

n<- nCa + nCo

J<- nCo/nCa # ratio of number controls to cases

q<- mean(Y)

q

library("ltmle")

library("SuperLearner")

library("ltmle")

library("tmle")

library("rpart")

library("gam")

library("splines")

library("foreach")

library("randomForest")

library("glmnet")

Q.SL.library <- c("SL.glm", "SL.step", "SL.glm.interaction", "SL.randomForest", "SL.gam", "SL.rpart", "SL.glmnet")

g.SL.library <- c("SL.glm", "SL.step", "SL.glm.interaction", "SL.randomForest", "SL.gam", "SL.rpart", "SL.glmnet")

R <- ltmle(

data = data,

Anodes="A",

Lnodes = NULL,

Cnodes = NULL,

Ynodes="Y",

abar=list(1, 0),

Qform=c(Y="Q.kplus1 ~ Parity + age + first_pregnancy + first_marriage + Breastfeding2 + marital_stat + education + occupation + A"),

gform="A ~ Parity + age + first_pregnancy + first_marriage + Breastfeding2 + marital_stat + education + occupation",

SL.library = list(Q = Q.SL.library, g = g.SL.library),

variance.method = "ic",

gcomp = FALSE,

iptw.only = FALSE,

observation.weights = as.vector(Wt),

id = NULL

)

print(summary(R, estimator="tmle"))

# Extract information from the ltmle R object

Q <- as.data.frame(R$Qstar)

EY0 <- mean(Q[,2]);EY0

EY1 <- mean(Q[,1]);EY1

ATE <- mean(Q[,1]-Q[,2]);ATE

RR <- EY1/EY0; RR

################################################################################

# Excess Risk (Boostraping for 95%CI)

################################################################################

ER <- mean(1 - Q[,2]/Q[,1]);ER

library(boot)

ER.w= function(Q,indices)

{

dat=Q[indices,]

(1 - (dat$V2)/(dat$V1))

}

# Can get original estimate, by plugging in indices 1:n

ER.w(Q,indices=1:nrow(Q))

# Draw 10000 bootstrap sample estimates

boot.out=boot(Q,ER.w,10000)

# Compute confidence intervals using percentile method

boot.ci(boot.out,type="perc",conf=0.95)

ER

#############################################################

# Population Attributable Fraction (Boostraping for 95%CI)

#############################################################

ATF <- mean(pec * (1 - Q[,2]/Q[,1])); ATF

library(boot)

ATF.w= function(Q,indices)

{

dat=Q[indices,]

pec * (1 - (dat$V2)/(dat$V1))

}

# Can get original estimate, by plugging in indices 1:n

ATF.w(Q,indices=1:nrow(Q))

# Draw 10000 bootstrap sample estimates

boot.out=boot(Q,ATF.w,10000)

# Compute confidence intervals using percentile method

boot.ci(boot.out,type="perc",conf=0.95)

ATF

**####End of Codes####**
